# COVID-19 Clinical Trials: Unraveling a Methodological Gordian Knot

**DOI:** 10.1164/rccm.202005-1942ED

**Published:** 2020-09-01

**Authors:** Alexander G. Mathioudakis, Markus Fally, Rola Hashad, Sean Knight, Timothy Felton, Jørgen Vestbo

**Affiliations:** ^1^ Manchester University NHS Foundation Trust Manchester Academic Health Science Centre Manchester, United Kingdom; ^2^ Division of Infection, Immunity and Respiratory Medicine The University of Manchester Manchester, United Kingdom; ^3^ Section for Pulmonary Diseases Herlev Gentofte Hospital Hellerup, Denmark and; ^4^ Faculty of Medicine Alexandria University Alexandria, Egypt

The coronavirus disease (COVID-19) pandemic is driving an unprecedented international research mobilization aiming to understand the disease and prevent an ongoing global health disaster (1). Within just 6 months, a record of more than 23,000 publications focusing on COVID-19 have been indexed in PubMed, while more than 2,150 research studies have been registered with the ClinicalTrials.gov database. This corresponds to approximately 13% of all registered studies during the same period. More than 1,200 interventional trials project to recruit more than 2 million participants. These figures highlight an astounding promptitude of the research community but also raise some concerns and methodological riddles.

The COVID-19 trial portfolio competes for proportions of the same patient population, which is projected to shrink over the coming months. This could limit individual trials’ recruitment rates and delay the revelation of urgently needed safety and efficacy results to inform clinical practice. The “collaborative” approach adopted in many trials that allows for coenrollment of patients into more than one controlled trial in the course of their illness requires close communication between trial teams and a robust analysis plan to minimize the risk of introducing bias.

The evaluation of an intervention in numerous independent trials running in parallel is far from ideal. Chloroquine or hydroxychloroquine are included as interventions in 178 registered trials, with a median recruitment target of 392 participants (range, 7–55,000). In contrast to registration trials, in which a pharmaceutical company has an overview of ongoing projects and access to interim results, most of these trials are led by academics who have limited awareness of the other trials’ progress and findings. As a result of this data fragmentation, a significantly larger number of patients will need to receive experimental interventions before their efficacy and safety profiles can be confirmed. In brief, this is neither effective nor ethical.

Delays in the accumulation of conclusive evidence deprives an immensely higher number of patients of potentially lifesaving treatments. In parallel, this duplication of effort leads to overuse of precious research funding and resources, potentially for very limited benefit. Once conclusive evidence on the efficacy of an intervention is acquired, many of these trials will have to discontinue early, after spending extensive resources, without adding much to the evidence base.

Because all these issues are obvious to any clinical researcher, or any keen thinker, one would expect global collaboration to conduct a small number of large, well-designed trials. One example hereof is the Solidarity trial, a pragmatic randomized controlled trial with an adaptive design organized by the World Health Organization, evaluating the most promising interventions. However, there are several reasons for conducting multiple trials. First, organizing multinational controlled clinical trials is often challenging as there are different legal requirements, procedures, and data sharing limitations. Second, the COVID-19 pandemic progressed too rapidly to allow for an international coordinated effort. Individual study funding limitations, politics, personal ambition, and pharmaceutical industry interests should also be mentioned here.

On the other hand, there are good clinical reasons for conducting more than one trial for the same intervention. In the course of COVID-19, there are several potential indications for pharmacotherapy (i.e., prevention as well as treatment of early disease, hospitalized patients, patients being admitted to the ICU, or those developing specific complications, such as the cytokine storm syndrome or myocarditis). Interventions may exert different behavior for different indications. Our experience from influenza suggests that antiviral treatments are likely to be more effective when administered earlier in the course of the disease (2). On the other hand, the risk of administering antiinflammatory treatments early in the course of a viral infection, such as COVID-19, is likely disproportionate to the anticipated benefit (3). Therefore, patients’ safety, resources, and cost implications need to be considered before recommending earlier interventions. Thus, we need safety and efficacy data of the administration of interventions at different time points in the disease course. Furthermore, the susceptibility to the virus and treatment response may vary across different populations, justifying the conduct of trials in more focused patient populations.

For all these reasons, the research community is now facing a challenging situation. There is a need to carefully manage the fragmented research data being collected to safely and swiftly draw conclusions. To achieve that, many of the larger trials plan interim analyses, that would offer preliminary data on the safety and efficacy of the interventions. In parallel, multiple living meta-analyses and guidelines are being set up to capture and combine trial reports as soon as they are released. To facilitate combination of emerging results in a meta-analysis, a core outcome set for COVID-19 trials is being developed, aiming to homogenize the outcomes that are evaluated and reported (ongoing).

Under normal circumstances, these approaches covering the standard spectrum of evidence-based methods would suffice. However, the daily death toll and incidence of COVID-19 call for an optimization of available methodologies. Moreover, waiting for each trial to report interim or final results may be counterintuitive, given the vast number of trials evaluating the same intervention in parallel. We suggest that interim data meta-analyses (or network meta-analyses) powered to evaluate key outcomes should be organized, as they could minimize the time needed to identify effective treatments and trigger their early introduction to routine care (Figure 1). However, this will be a major undertaking, requiring global, multidisciplinary coordinated efforts. Live recruitment progress updates of all trials should be shared within a core meta-analysis group. Trialists will need to agree to share interim data once the overall studied population will achieve the required power, and this should be supported and/or requested by the regulatory boards, ethics committees, and funders. In addition, researchers will need to be reassured that publication of preliminary data from their studies will not prevent or downgrade their final publications. Oversight by an independent international scientific organization such as the European Respiratory Society, the American Thoracic Society, or the World Health Organization would be beneficial.

**Figure 1.**
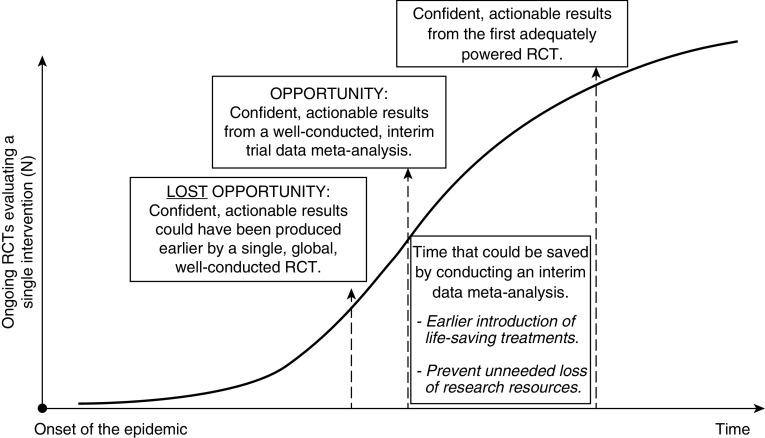
Graphical representation of the timeline to the acquisition of confident, actionable results regarding the safety and clinical effectiveness of clinical interventions for coronavirus disease (COVID-19) infection, demonstrating the benefits of conducting an interim data meta-analysis. RCT = randomized controlled trial.

A major challenge when conducting such a meta-analysis would be for the authors to ensure the accuracy, validity, and transparency of the interim data reported by individual, ongoing trials. This issue is further reinforced by recent controversies in published data that led to the retraction of several studies on COVID-19 by major scientific journals, including *The New England Journal of Medicine* and *The Lancet*. A simple solution for this issue would be for members of the steering committees of the ongoing trials to be part of the research and authoring team, to vouch for the integrity of interim results of their trials. This approach would enhance the confidence in the integrity of the data and will also encourage trialists to contribute their data to such meta-analyses. In addition, acknowledgment of the trialists’ contribution is only appropriate, because such meta-analyses would pose additional work to the trial team, which will have to review and “clean” interim data to confirm their accuracy. Systematic reviewers should also consider focusing their meta-analysis on a small number of outcomes that are important to patients, simple to measure and objective, such as mortality, as it is more likely that the trialists will be able to deliver accurate interim data.

Strategic decisions about the trials’ conduct in response to emerging evidence will also be challenging. Lack of efficacy of an intervention should lead to the discontinuation or repurposing of all other trials evaluating the same intervention. In parallel, once the efficacy of any treatment is confirmed, all placebo or standard care arms of all other trials assessing the same treatment indication will need to be reevaluated to avoid depriving patients of effective treatments. In view of the numerous ongoing trials, clinical trialists will need to be very vigilant to identify early emerging data. For example, a recent preliminary report from the ACTT-1 (Adaptive COVID-19 Treatment Trial) double-blinded clinical trial suggested that remdesivir could decrease the duration of hospital stay by 4 days for people admitted to the hospital because of COVID-19 infection (4). In addition, the RECOVERY (Randomised Evaluation of COVID-19 Therapy) pragmatic, open-label, randomized clinical trial group in a press release disclosed preliminary findings regarding dexamethasone and hydroxychloroquine, based on data from 2,000 patients that received each of these interventions and 4,000 patients that received placebo. Based on these data, dexamethasone appears to decrease the mortality by one-fifth in patients with COVID-19 requiring oxygen therapy and by one-third among ventilated patients, whereas hydroxychloroquine did not appear to confer any clinical benefits. These findings should trigger a review of the ongoing trials’ interventions and designs. It is crucial that funders, ethics committees, and regulatory bodies should encourage trialists to include modification plans for trials whose conduct is deemed unnecessary or inappropriate.

Overall, in the context of the COVID-19 pandemic, it was unavoidable to launch multiple controlled clinical trials, often evaluating the same intervention. For this reason, we now need to develop strategies and methodologies that will allow us to make the best use of the data being collected, while protecting the patients from disadvantageous outcomes.

## Supplementary Material

Supplements

Author disclosures
