# Supplementary material for: COVID-19 Clinical Trials: Unraveling a Methodological Gordian Knot
Source: Am J Respir Crit Care Med. 2020 Sep 1;202(5):635–7. doi: 10.1164/rccm.202005-1942ED (PMC7462407; doi:10.1164/rccm.202005-1942ED)
Supplement: Supplements [file rccm.202005-1942ED.html]

COVID-19 Clinical Trials: Unraveling a Methodological Gordian Knot | American Journal of Respiratory and Critical Care Medicine

- disclosures.pdf (200 KB)
